# Developing and validating a nomogram prediction model for osteoporosis risk in the UK biobank: a national prospective cohort

**DOI:** 10.1186/s12889-025-22485-x

**Published:** 2025-04-03

**Authors:** Xinning Tong, Shuangnan Cui, Huiyong Shen, Xiaoxin Iris Yao

**Affiliations:** 1 Department of Orthopaedics, The Eighth Affiliated Hospital, Sun Yat-Sen University, 3025 Shennan Road, Shenzhen, 518033 China; 2 Department of Epidemiology, School of Public Health, Sun Yat-sen University, Guangzhou, China; 3 Department of Clinical Research, The Eighth Affiliated Hospital, Sun Yat-sen University, Shenzhen, China; 4 Guangdong Provincial Clinical Research Center for Orthopedic Diseases, The Eighth Affiliated Hospital, Sun Yat-sen University, Shenzhen, China

**Keywords:** Nomogram prediction model, Osteoporosis, Risk identification, Disease prevention

## Abstract

**Background:**

Osteoporosis is a prevalent bone disease that increases frailty. Developing a nomogram prediction model to predict osteoporosis risk at multiple time points using bone mineral densities, behavioral habits, and clinical risk factors would be essential to identify individual risk and guide prevention.

**Methods:**

The study population from the UK Biobank was followed from 2014 to December 31st, 2022. The study outcome was identified as the first occurrence of osteoporosis in the UK Biobank during the follow-up period. After rebalancing with the synthetic minority over-sampling technique, a nomogram prediction model was developed using a LASSO Cox regression. Model discrimination between different risk levels was visualised with Kaplan-Meier curves, and model performance was evaluated with integrated c-index, time-dependent AUC, calibration curves and decision curve analysis (DCA).

**Results:**

The model identified several risk factors for osteoporosis, including higher age, underweight, and various clinical risk factors (such as menopause, lower hand grip strength, lower bone mineral density, fracture history within 5 years, and a history of chronic disease including hypercholesterolemia, cardiovascular disease, bone disease, arthritis, and cancer). Kaplan-Meier curves showed that risk levels predicted by the nomogram model were significantly distinct. The c-indexes were 0.844 and 0.823 for training and validation datasets, respectively. Time-dependent AUC, calibration curves and DCA indicated good discrimination, model fit and clinical utility, respectively.

**Conclusions:**

The nomogram model could properly quantify the five-year risk of osteoporosis and identify high-risk individuals. This might effectively reduce the burden of osteoporosis on the population.

**Supplementary Information:**

The online version contains supplementary material available at 10.1186/s12889-025-22485-x.

## Introduction

Osteoporosis is a prevalent and chronic bone disease, characterized by increased bone fragility and diagnosed through a dual-energy X-ray absorptiometry (DXA) T-score of bone mineral density ≤ -2.5 [1–3]. It significantly raises the risk of osteoporotic fractures, which can cause pain, limit mobility, decrease independence, and reduce quality of life. These fractures also contribute to higher mortality rates and impose a substantial economic burden [4].

As a tool to quantify risks and benefits, risk prediction models can provide more objective and accurate information for patients, doctors, and health managers to make decisions [5, 6]. The risk prediction model of osteoporosis has an important potential application for the health management of osteoporosis patients. Predictive models can give patients the probability of developing osteoporosis in the future, providing more objective evidence for early intervention [7–9]. Machine learning methods have been used to develop prediction models. However, the complex relationships obtained from the machine learning algorithms between risk factors and osteoporosis risk pose challenges for clinical intervention [7]. Traditional regression methods are more suitable for clinical use as they detect explainable factors. However, existing prediction tools, such as the Fracture Risk Assessment Tool (FRAX), Qfracture, and Garvan Fracture Risk Calculator (Garvan) [10–13] were designed to identify individuals with a high risk of fractures within a long-term timeframe (e.g. 10 years). One widely accepted prediction tool for osteoporosis, the Osteoporosis Self-assessment Tool (OST), has limitations in its external applicability [14, 15]. These prediction models have mainly been developed using homogeneous female populations and lack diversity in risk factors. There is a gap in the research regarding prediction models that can identify individuals at risk of osteoporosis among the general population, including those without osteoporotic fractures. To broadly reduce the burden of osteoporosis from the public health perspective, it is essential to develop prediction models to accurately assess an individual’s risk of developing osteoporosis over a moderate time period.

This study aims to develop a comprehensive prediction model for osteoporosis, considering multiple bone mineral density (BMD) sites and various clinical risk factors. The model was developed as a user-friendly nomogram to assist healthcare professionals in assessing individual risk. The benefits of this osteoporosis risk prediction model would be reflected in every link of the tertiary prevention system of the disease. With the help of non-invasive, low-cost, and easy-to-collect indicators, the model will provide a personalised assessment of risk factors, enabling targeted interventions to prevent or delay the onset of osteoporosis, ultimately improving the clinical management of osteoporosis.

## Methods

### Study population

The UK Biobank, a national-wide prospective cohort, was utilised as the data source for our study [16]. This electronic database comprises over 500,000 participants aged 40–69 who were recruited from across the United Kingdom between 2006 and 2010. During an initial assessment visit, participants provided detailed information on demographic characteristics, lifestyle habits, and medical history through questionnaires. In addition, physical measurements and biological samples were collected. The UK Biobank also offers longitudinal health outcome data through multi-source data linkage. As part of a follow-up assessment conducted in 2014 afterward, approximately 50,000 participants underwent DXA scans to measure bone mineral density at various skeletal sites. We followed participants from their DXA scan date until the earliest occurrence of an osteoporosis diagnosis, death, loss to follow-up, or the end of the observation period on December 31st, 2022, whichever came first. Participants with a history of osteoporosis and participants with a femur neck or lumbar spine T-score less than − 2.5 at the time of recruitment were excluded [2, 3].

### Outcomes and potential candidate variables

We identified the first occurrence of osteoporosis through the International Classification of Disease-10 (ICD-10), M80-M82, from all available health record sources linked by the UK Biobank, including primary care data, hospital inpatient data, death register records, and self-reported medical conditions during the UK Biobank center visits. We identified potential predictor variables for the nomogram prediction model based on literature reviews evaluating known risk factors for poor bone health and osteoporosis [7–13, 17–20]. Variables in the UK Biobank dataset aligned with established risk factors from prior research were prioritised. Bone mineral density measurements from different sites were tested for collinearity, and final skeletal sites incorporated into the model were selected in consultation with clinicians. Any variables with missing data rates exceeding 70% (according to data distribution) were excluded, and participants without complete data on all candidate predictors were also excluded from the analysis. The comparison of participants with and without complete candidate predictors was shown in eTable 1, where a standardised mean difference less than 0.2 was considered a small difference between two groups [21, 22].

### Nomogram development

To reflect the real-world data and allow us to improve the model performance, we randomly selected a subset of participants who did not develop osteoporosis during the follow-up period and combined them with participants with osteoporosis during the follow-up period in a 6:1 ratio of the two groups [23–25]. We then divided this dataset using a 70/30 split, allocating 30% of the subjects to the validation dataset. The remaining 70% of the subjects, along with the remaining participants without osteoporosis from the previous step, were assigned to the training dataset. An illustration of the dataset preparation for model development and validation was shown in eFigure 1.

We employed the synthetic minority over-sampling technique (SMOTE), one popular method to solve the data imbalance problem, to address class imbalance issues before developing the model [26, 27]. No information will be lost during this process, and overfitting problems from simple copying samples will be avoided [27]. By applying SMOTE, we aimed to produce a resampled dataset with a more balanced distribution of outcome classes to improve model performance and generalizability during subsequent predictive modelling.

Within the training dataset, we utilised the least absolute shrinkage and selection operator (LASSO) Cox regression to identify significant predictors and construct the prediction model. We optimised the tuning parameter lambda using 10-fold cross-validation and chose the largest value of lambda as within one standard error (lambda.1se) of the cross-validated error for lambda min to ensure variable shrinkage and minimise overfitting. Only variables with non-zero coefficients after LASSO regularisation were retained for further analysis.

We developed the model as an individualised nomogram incorporating the key risk factors identified through the LASSO process. Nomograms visually represent an individual’s predicted risk score based on their unique combination of clinical attributes. By incorporating the LASSO-selected variables, our model calculates 2-year, 3-year, and 5-year osteoporosis probabilities for individuals.

### Evaluation of model performance

We considered four criteria for evaluating the model performance in the balanced training datasets and validation datasets. Firstly, we stratified participants into three risk groups according to the tertiles of their nomogram-predicted scores. We then used Kaplan-Meier survival curves and the log-rank test to compare osteoporosis-free survival across these risk tiers. Secondly, we calculated the concordance index (c-index) to assess the model’s ability to distinguish between observed and predicted osteoporosis probabilities. We also generated area under the curve (AUC) values from time-dependent receiver operating characteristic (ROC) curves at two, three-, and five-years post-baseline. Thirdly, we used a calibration curve to evaluate the model’s goodness of fit. This visualisation assesses the consistency between predicted and actual osteoporosis risk at five years. Ideally, the calibration plot would show perfect predictive accuracy with points closely aligned along the 45-degree reference line and intercepts of zero [28]. Lastly, we did a decision curve analysis (DCA) to test the clinical usefulness of the model. The predictive ability of the nomogram was also compared with the Osteoporosis Self-Assessment Tool (OST) by c-index, calibration plot and DCA model.

We reported the continuous variables as means with standard deviations (SD) or median with interquartile range (IQR) and reported categorical variables with frequencies and percentages. A two-sample t-test (continuous variables) or χ^2^ tests (categorical variables) was used to test differences between the training and validation datasets. All data analyses were performed with R software (version 4.3.1, R Foundation for Statistical Computing, Vienna, Austria). R packages *‘survival’*,* ‘rms’*, and *‘hdnom’* were mainly used for model development and validation.

## Results

### Characteristics of the training and validation datasets

**Fig. 1:**
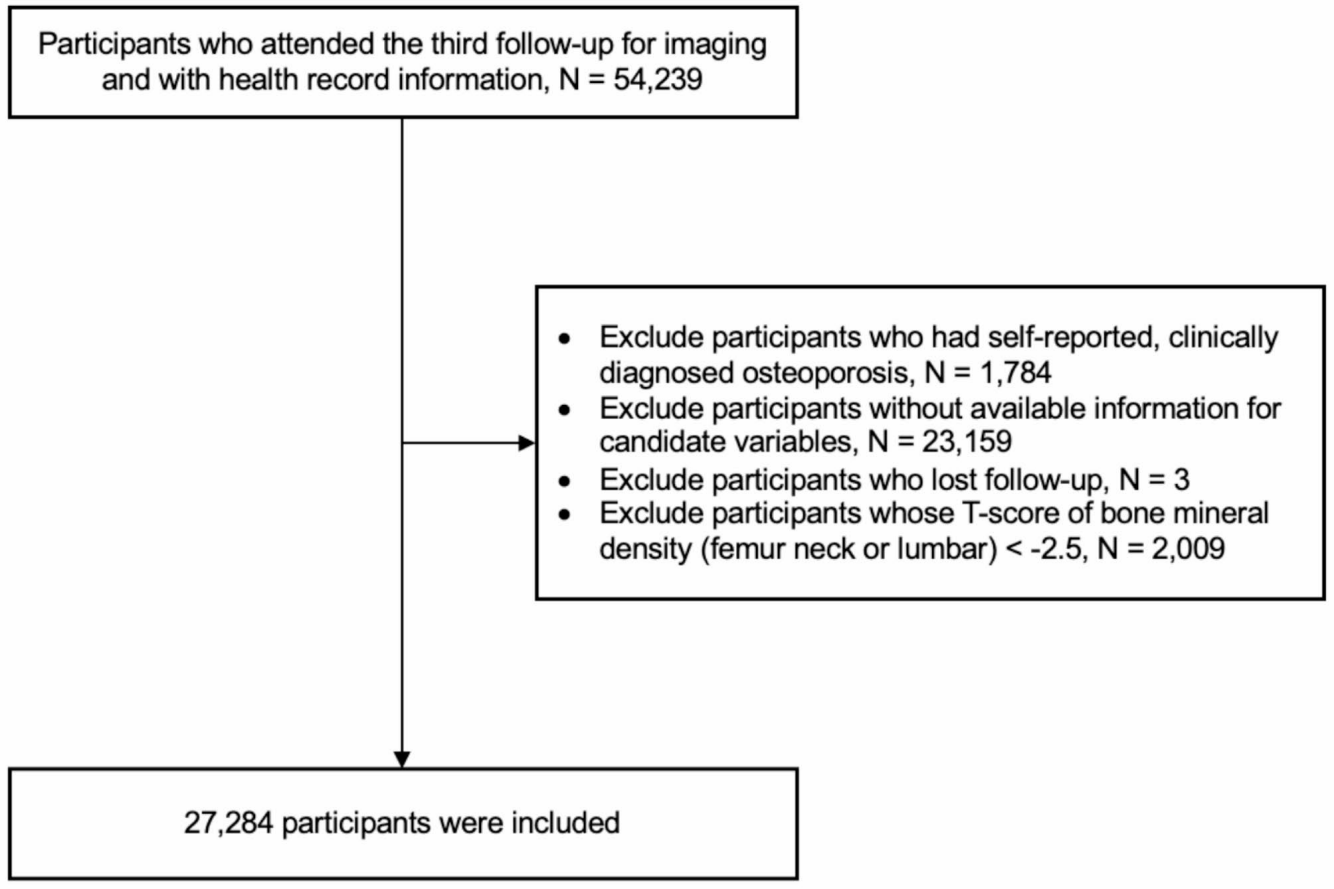
Flowchart of subject selection

A total of 27,284 participants with complete information on candidate variables were included. The flowchart of participant selection is shown in Fig. 1. No significant difference was observed between the participants before and after excluding people without complete candidate predictors (eTable 1). During a median of 5.03 (IQR: 4.11–6.55) years follow-up period, we observed 194 incident osteoporosis. We used 34,764 rebalanced observations to develop the prediction model and used 407 observations for model validation. The characteristics of the participants in the training and validation datasets are shown in Table 1. The mean age was 63.60 years (SD: 7.28) for the training dataset and 63.54 (8.06) for the validation dataset, with 44.7% and 45.3% women, and 92% and 94% White ethnicity, respectively. Both cohorts had around 73% of households had an annual income before tax of lower than £51,999. The percentage of participants with university or college degrees was similar between the training (46.3%) and validation datasets (48.4%). We observed that approximately 35% and 33% of participants in training and validation datasets had a standard range of body mass index (BMI), respectively. Moreover, 4% and 3% were current smokers, and 17% and 18% were daily drinkers for the training and validation datasets, respectively. Additionally, we found that the average bone mineral density measured at the heel bone, arm, lumbar and femur, prevalence of fracture history within five years, and prevalence of various self-reported clinical risk factors, including diabetes, diabetic medication (insulin), hypertension, blood pressure medication, high cholesterol, cholesterol lowering medication, cardiovascular disease, bone disease, arthritis, and cancer were all similar between the two cohorts.

**Table 1:** Characteristics of the training and validation datasets

	Training	Validation	*P* value
	34,764	407	
Ethnicity			
White	31,894 (91.7)	381 (93.6)	0.203
Non-white	2870 (8.3)	26 (6.4)	
Sex			0.842
Female	19,003 (44.7)	225 (45.3)	
Male	15,761 (45.3)	182 (44.7)	
Age, years, Mean (SD)	63.60 (7.28)	63.54 (8.06)	0.861
Household income (%)			0.007
Less than £30,999	13,947 (40.1)	189 (46.4)	
£31,000 to £51,999	11,489 (33.0)	106 (26.0)	
Greater than £52,000	9328 (26.8)	112 (27.5)	
Qualification (%)			0.034
Other	6327 (18.2)	90 (22.1)	
GCSEs or equivalent	6838 (19.7)	72 (17.7)	
A-levels or equivalent	5491 (15.8)	48 (11.8)	
University or college	16,108 (46.3)	197 (48.4)	
Body mass index (%)			< 0.001
Normal (18.5 to < 25 kg/m^2^)	12,303 (35.4)	134 (32.9)	
Underweight (< 18.5 kg/m^2^)	2014 (5.8)	5 (1.2)	
Overweight or obesity (25 kg/m^2^ or higher)	20,447 (58.8)	268 (65.8)	
Daily drinker (%)	5953 (17.1)	74 (18.2)	0.619
Current smoker (%)	1343 (3.9)	12 (2.9)	0.410
Menopause (%)	2798 (8.0)	35 (8.6)	0.753
Hand grip strength (%)	28.21 (10.2)	27.91 (10.4)	0.555
Bone mineral density (g/cm^2^), Mean (SD)			
Heel bone	0.54 (0.13)	0.54 (0.14)	0.771
Arm	0.92 (0.14)	0.93 (0.14)	0.426
Lumbar	1.18 (0.18)	1.20 (0.18)	0.152
Femur	0.93 (0.13)	0.94 (0.14)	0.080
Fracture history within 5 years (%)			0.002
None	30,502 (87.7)	369 (90.7)	
Trunk	1385 (4.0)	2 (0.5)	
Other	2877 (8.3)	36 (8.8)	
Diabetes (%)	2033 (5.8)	26 (6.4)	0.722
Insulin (%)	251 (0.7)	3 (0.7)	1.000
Hypertension (%)	10,534 (30.3)	132 (32.4)	0.381
Blood pressure medication (%)	7941 (22.8)	110 (27.0)	0.053
Hypercholesterolemia	9376 (27.0)	126 (31.0)	0.081
Cholesterol lowering medication (%)	7984 (23.0)	105 (25.8)	0.197
Cardiovascular disease (%)	1934 (5.6)	27 (6.6)	0.408
Bone disease (%)	972 (2.8)	6 (1.5)	0.144
Arthritis (%)	6723 (19.3)	82 (20.1)	0.728
Cancer (%)	4912 (14.1)	60 (14.7)	0.779

SD: standard deviation

### Model-identified risk factors

We initially considered 25 candidate risk factors for inclusion in the model. To evaluate categorical variables during the least absolute shrinkage and selection operator (LASSO) selection process, we encoded them as dummy variables, resulting in 30 transformed variables. After applying LASSO regression, six risk factors, including gender, daily drinker, current smoker, disease history of diabetes, usage of insulin and cholesterol lowering medication were excluded from the final prediction model.

**Fig. 2:**
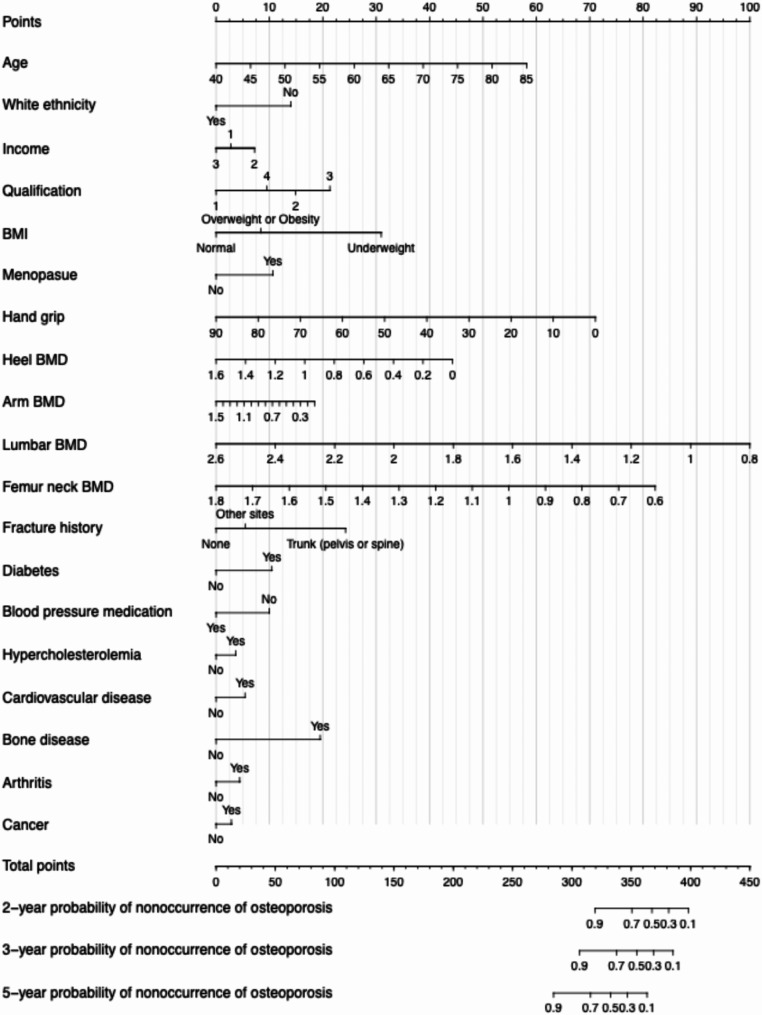
Two, three, and five-year risk score by nomogram prediction models for osteoporosis. Note: Income: 1, Less than £30,999; 2, £31,000 to £51,999; 3, Greater than £52,000. Qualification: 1, Other; 2, GCSEs or equivalent; 3, A-levels or equivalent; 4, University or college. BMI: body mass index [Normal (18.5 to < 25 kg/m^2^); Underweight (< 18.5 kg/m^2^); Overweight or Obesity (25 kg/m^2^ or higher)]; BMD: bone mineral density, g/cm^2^

The hazard ratio (HR) and 95% confidence interval (CI) for each included variable are shown in eTable 2. Several factors were identified as protective indicators associated with a lower likelihood of developing osteoporosis. These included white ethnicity, higher hand grip strength, greater bone mineral density at various sites (heel, arm, lumbar, femur), hypertensive medication. Conversely, factors such as higher age, underweight, menopause, recent history of fractures, and self-reported disease histories of hypercholesterolemia, cardiovascular disease, bone disease, arthritis, and cancer were associated with an increased risk of developing osteoporosis. A nomogram prediction model was developed using the variables selected by the LASSO regression to visualise the individual predicted risk scores of osteoporosis at 2-year, 3-year, and 5-year time points (Fig. 2). Each risk factor corresponds to a point value on the scale, and the total risk by adding up the points from all applicable factors. This allows quantification and comparison of osteoporosis risk on an individual level.

### Performance of the prediction model

**Fig. 3:**
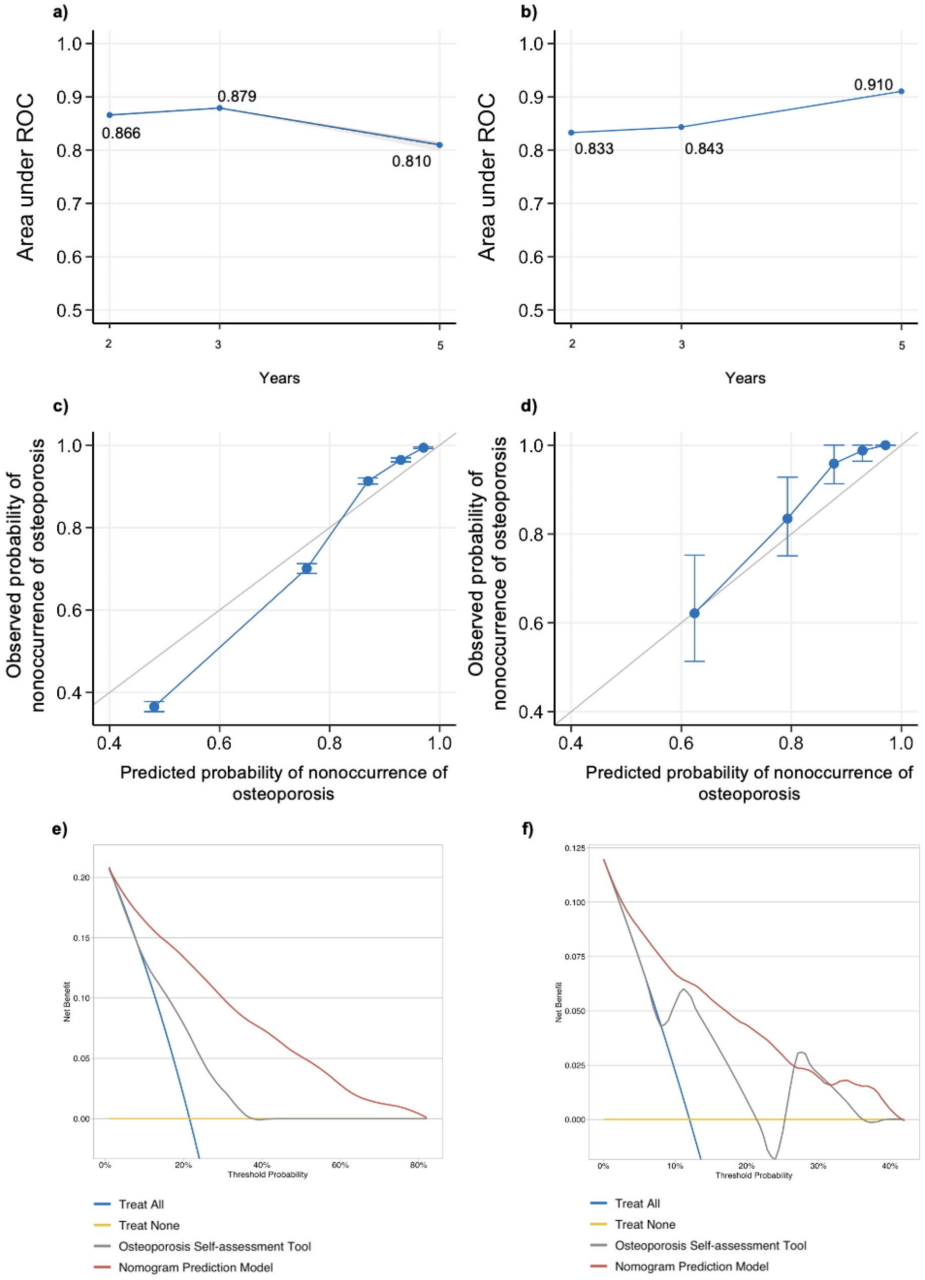
Performance of the nomogram model for risk of osteoporosis. Note: **a**) Time-dependent area under the curve (AUC) values from receiver operating characteristic (ROC) for the training cohort. Values represent the mean value of AUC at time points 2-year, 3-year, and 5-year. **b**) Time-dependent AUC for the validation cohort. Values represent the mean value of AUC at time points 2-year, 3-year, and 5-year. **c**) Calibration of the nomogram model in the training cohort. **d**) Calibration of the nomogram model in the validation cohort. **e**) Decision Curve Analysis (DCA) of the nomogram model and the osteoporosis self-assessment tool in the training cohort. **f**) Decision Curve Analysis (DCA) of the nomogram model and the osteoporosis self-assessment tool in the validation cohort

The integrated c-index of the model is 0.844 (95% CI: 0.840, 0.848) for the training dataset and 0.823 (95% CI: 0.776, 0.870) for the validation dataset. The time-dependent AUC and calibration curves of the prediction model performance are shown in Fig. 3. The average time-dependent AUCs in the second year, third year, and fifth year are 0.866, 0.879, and 0.810, respectively, for the training dataset [Figure 3 (a)] and 0.833, 0.843, and 0.910, respectively, for the validation dataset [Figure 3 (b)]. The calibration figures showed the model had a good calibration performance as the predicted line is close to the 45-degree line, which means the predicted probabilities of osteoporosis nonoccurrence are approximate to the observed situations [Figure 3 (c) and (d)].

**Fig. 4:**
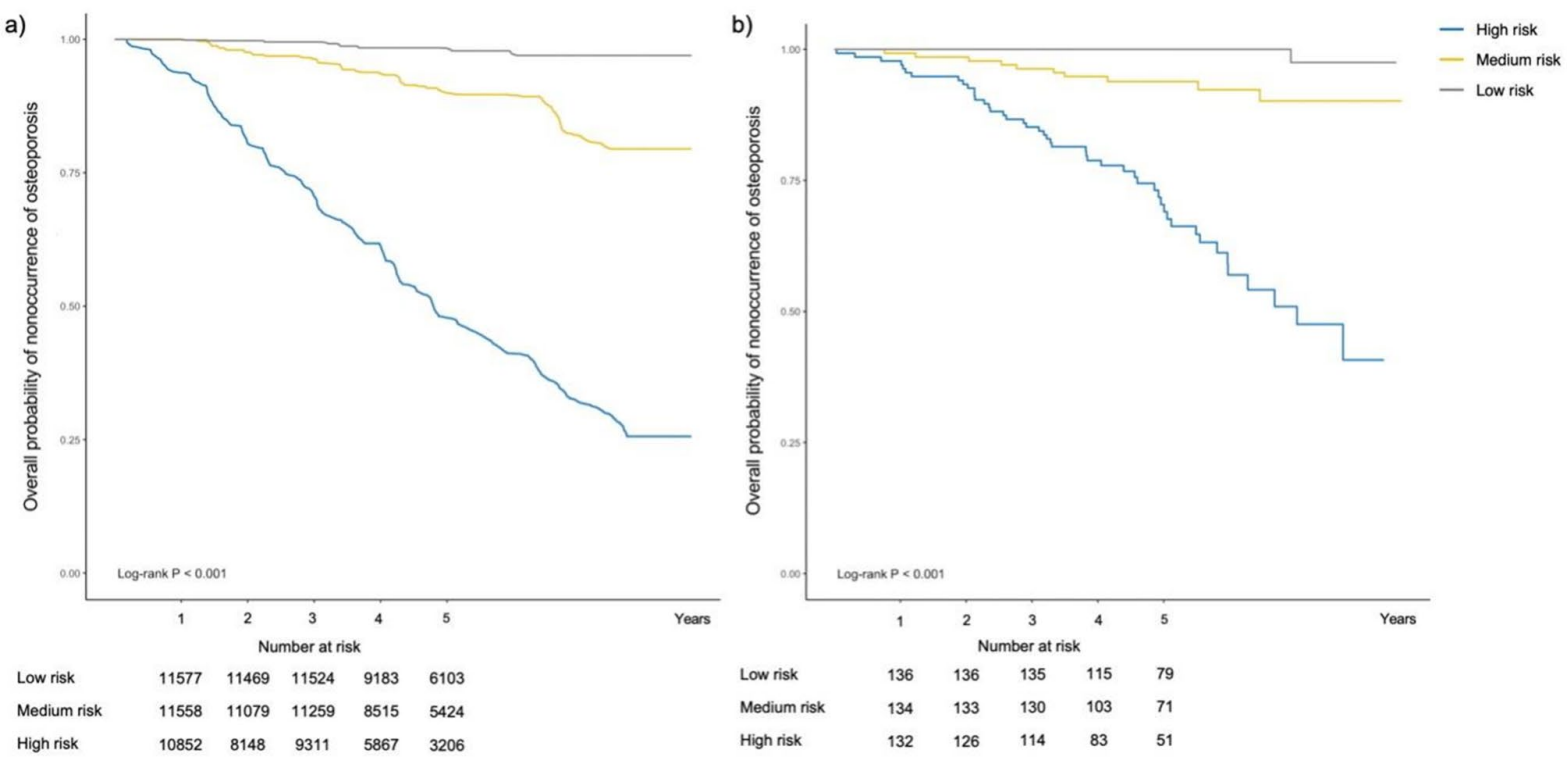
Kaplan-Meier survival curves by nomogram-predicted osteoporosis risk strata. Note: (**a**) Training dataset; (**b**) Validation dataset

Figure 4 shows a Kaplan-Meier survival curve comparing osteoporosis-free probability over time based on tertiles of risk scores predicted by the nomogram. The model could significantly differentiate the osteoporosis-free probability between low, median and high-risk subjects (log-rank test, *P* < 0.001) in training and validation datasets. Participants predicted to be in a higher-risk group for osteoporosis would face lower osteoporosis-free probabilities throughout the follow-up period.

C-indexes of the Osteoporosis Self-Assessment Tool (OST) in the training and validation sets were 0.669 (95% CI: 0.663, 0.675) and 0.797 (95% CI: 0.748, 0.846), respectively, which were lower than those of our nomogram model. Calibration curves showed that OST performed poorer than our model (eFigure 2). The results of the decision curve analysis [Figure 3 (e) and (f)] demonstrated that our nomogram offered a greater net benefit compared to the OST self-assessment tool. This indicated that our model has an efficient predictive ability in predicting the 2-year, 3-year, and 5-year risk of osteoporosis.

## Discussion

This study introduced an innovative approach by incorporating four sites’ bone mineral density and various clinical risk factors and lifestyle behaviours to develop a nomogram prediction model for quantifying osteoporosis risk at multiple time points. The model identified modifiable risk factors, shedding light on their impact on osteoporosis development. This model has the potential to facilitate early identification of at-risk individuals and the implementation of targeted preventative strategies. With good predictive accuracy, well-accepted calibration performance, and satisfactory clinical net benefit, the model could serve as an efficient tool for early osteoporosis identification and intervention strategies [29].

Our model identified higher age, underweight BMI, menopause, lower hand grip strength, lower bone mineral density, fracture history within 5 years, and a history of chronic disease including hypercholesterolemia, cardiovascular disease, bone disease, arthritis, and cancer, as risk factors for osteoporosis. The risk factors identified by our nomogram model were largely consistent with those shown in observational studies and well aligned with those variables incorporated into several osteoporosis and fracture prediction tools [8, 13, 30].

Bone mineral density (BMD) can vary across different body sites. A population-based DXA study found that the peak BMD values and rate of BMD loss with age differed across various sites, suggesting that BMD in different areas may not have the same impact on osteoporosis risk [31]. Our research found that the loss of BMD in the lumbar neck region contributed the most to the increased risk of osteoporosis, followed by femur BMD, and heel BMD. These findings suggest that the impact of BMD loss on osteoporosis risk can vary across different body sites. The mechanisms behind this are not fully clear but require further exploration.

Our model found that non-white ethnicity people showed a higher risk of osteoporosis compared to White ethnic participants. This finding diverged from global patterns of osteoporosis prevalence observed in a meta-analysis, which exhibited that the European population had the highest prevalence rate of osteoporosis than other areas’ populations [32]. However, given that our cohort was racially homogeneous (over 90% were white), it is plausible that this lack of ethnic diversity constrained the explanatory power of the race variable within our specific study population. Future research cohorts incorporating more heterogeneous ethnicities will be necessary to validate the influence of ethnicity found here.

Our nomogram findings revealed interesting insights regarding specific socioeconomic dimensions. Surprisingly, participants with post-secondary education (e.g., GCSEs, A-levels, college/university degrees or their equivalent qualifications) appeared to have a higher risk of osteoporosis compared to those without at least a secondary degree. One possible explanation is that higher education may enable greater engagement with healthcare services, thereby increasing osteoporosis detection through routine screening [33, 34]. In other words, more educated individuals may have better health literacy and be more likely to learn of an osteoporosis diagnosis due to enhanced access to preventive care rather than having inherently higher underlying risk. Additionally, other demographic confounders, such as occupation and urban versus rural living conditions, may obscure the true relationships. For example, higher education levels may correspond with sedentary jobs [35], leading to lower physical activity levels and, consequently, increased osteoporosis risk.

The underlying mechanisms between low bone density and metabolism conditions such as cardiovascular disease (CVD), hypercholesterolemia, hypertension and diabetes are not yet fully understood. The LASSO analyses showed that a history of hypertension did not significantly improve the model’s performance and, therefore, was excluded from the prediction model. While the model did identify CVD, diabetes, and hypercholesterolemia are risk factors for osteoporosis. Several epidemiological studies have reported an association between osteoporosis and an increased risk of an irregular lipid profile or a higher risk of CVD [36–38]. Additionally, diabetes is associated with irregular lipid profiles that would increase osteoclast function but decrease osteoblast function, thereby increasing the risk of osteoporosis [39]. Our model revealed that people regularly use blood pressure medication, which was observed to be beneficial in reducing the risk of osteoporosis. It aligns with previous findings that medications targeting blood pressure could positively impact bone metabolism [40].

In our study, the presence of cancer was identified as a risk factor for osteoporosis. Previous studies have demonstrated that cancer survivors have an increased risk of bone mineral density loss and major osteoporotic fractures [41]. Several mechanisms may be responsible for these findings [42, 43]. The metastatic disease often targets the skeleton. Prostate cancer, breast cancer, and multiple myeloma have been identified as particularly associated with bone metastases and bone loss [44]. Furthermore, therapies used for specific cancers, such as prostate cancer and breast cancer, result in testosterone levels being reduced to castrate levels, which is a significant risk factor for bone loss and fractures [45]. Our nomogram model supports the view that bone health in cancer patients should be monitored.

The nomogram prediction model for osteoporosis offers significant contributions to public health through several aspects. Firstly, it enhances individual risk assessment for osteoporosis by integrating various factors, enabling healthcare providers to identify high-risk individuals even when their bone mineral density remains within normal limits. Secondly, this individualised prediction model facilitates the customisation of intervention strategies tailored to the specific needs of each patient. Such a personalised approach is likely to yield improved patient outcomes by ensuring that interventions, whether lifestyle modifications or pharmacotherapy, are precisely targeted. Finally, the model serves as an effective tool for engaging patients in their own health management. By delivering clear risk assessments, clinicians can educate patients about their osteoporosis risk factors, fostering a sense of ownership over their health and encouraging adherence to preventive measures.

There are several strengths of our nomogram prediction model for osteoporosis. First, our model predicts the risk of osteoporosis in the following years (e.g. 2, 3, or 5-year risk) and provides hints earlier than previous models that predicted fractures. Second, our model can be applied to the general population, which is broader than the previous model (special populations such as ageing people and women). Third, our model provides a user-friendly nomogram that will be more useful for screening, identifying, and intervening osteoporosis in clinical practice. Through targeted interventions suggested by the model, physicians can provide personalised recommendations to individuals at risk of osteoporosis to make disease prevention more effective. Fourth, distinct from existing osteoporotic fracture models using data derived from clinical trials (e.g. FRAX) or clinical records alone (e.g. QFracutrue) [8, 13], our study utilised comprehensive health screenings, encompassing a wide range of demographic profiles, lifestyle factors, comorbidities, and multiple-site bone mineral density assessments from a large general population sample (over 27 thousand participants). Last but not least, compared to those prediction models using machine learning methods, our nomogram prediction model can identify individual-specific modifiable risk factors for intervention and demonstrate comparable performance (AUCs around 0.8) [7].

This study had several limitations. Firstly, this model did not include time-varying information, and changes in lifestyle behaviour might accelerate or delay the onset of osteoporosis. However, the ability to predict future osteoporosis risk based on baseline information means that the model has the advantage of convenience, and the existing model has shown good performance in training and testing datasets. Secondly, the low prevalence of certain chronic diseases (less than 1%), such as chronic obstructive pulmonary disease, epilepsy, renal failure, thyroid problems, parathyroid problems and Parkinson’s disease, within our sample datasets resulted in their exclusion from the prediction model, potentially limiting its ability to predict osteoporosis risk in individuals with these conditions. Thirdly, our study was limited in its ability to ascertain the outcome of osteoporosis in individuals who did not undergo DXA assessments during the follow-up period. This potential bias may result in an underestimation of the risk of osteoporosis in our model. Nevertheless, the DCA results demonstrated that our model would provide clinical benefit across a broad range of threshold probabilities. Fourthly, our study used a cause-specific hazard model that did not consider competing risks (e.g., death from causes other than osteoporosis) because our cohort consisted of participants older than 45 years with a relatively low mortality rate (approximately 1%) and a short follow-up period (median 5.03 years) [46]. Lastly, our model was only validated within the same population with mostly White ethnicity, and external validation with more diverse populations is required in future studies to understand its generalizability.

## Conclusion

Our model offers a non-invasive and accessible approach to quantifying the risk of osteoporosis over various time frames among the general population. Our model recognises the relationship of socioeconomic status, existing comorbidities, and modifiable lifestyles with long-term osteoporosis risk. By following the lifestyle modifications recommended by our model, individuals at high risk can proactively improve their health outcomes and decrease their susceptibility to osteoporosis. Ultimately, it is expected to improve population health and reduce the disease burden of osteoporosis.

## Electronic supplementary material

Below is the link to the electronic supplementary material.

Supplementary Material 1

## Data Availability

Data sharing is not applicable to this article as UK Biobank data were used under license and thus not publicly available. Access to the UK Biobank data can be requested through a standard protocol (https://www.ukbiobank.ac.uk/enable-your-research/apply-for-access).
