# Supplementary material for: Developing and validating a nomogram prediction model for osteoporosis risk in the UK biobank: a national prospective cohort
Source: BMC Public Health. 2025 Apr 3;25:1263. doi: 10.1186/s12889-025-22485-x (PMC11970002; doi:10.1186/s12889-025-22485-x)
Supplement: Supplementary file 1 — Supplementary Material 1 [file 12889_2025_22485_MOESM1_ESM.docx]

**Supplementary material**

Developing and validating a nomogram prediction model for osteoporosis risk in the UK Biobank: a national prospective cohort

Xinning Tong, PhD^1^, Shuangnan Cui, Mphil^1^, Huiyong Shen, MD^1,2^, Xiaoxin I Yao, PhD^1,2*^

Content:

eTable 1. Characteristics of participants with and without complete candidate variables

eTable 2. Hazard ratios of individual parameters in the risk model for osteoporosis

eFigure 1. Illustration of dataset preparation for model development and validation

eFigure 2. Calibration of the osteoporosis self-assessment tool within the training and validation cohort

eTable 1. Characteristics of participants with and without complete candidate predictors

|  | All Subjects | Recruited Subjects | SMD |
| --- | --- | --- | --- |
|  | 54239 | 27284 |  |
| Ethnicity |  |  |  |
| White | 50026 (92.3) | 25255 (92.6) |  |
| Non-white | 4196 (7.7) | 2029 (7.4) | 0.011 |
| Sex |  |  |  |
| Female | 28035 (51.7) | 13083 (48.0) | 0.075 |
| Male | 26204 (48.3) | 14201 (52.0) |  |
| Age, years, Mean (SD) | 64.54 (7.81) | 62.95 (7.58) | 0.206 |
| Household income (%) |  |  |  |
| Less than £30,999 | 19368 (39.9) | 10563 (38.7) | 0.024 |
| £31,000 to £51,999 | 14714 (30.3) | 8372 (30.7) |  |
| Greater than £52,000 | 14518 (29.9) | 8349 (30.6) |  |
| Qualification (%) |  |  |  |
| Other | 11699 (21.8) | 5454 (20.0) | 0.049 |
| GCSEs or equivalent | 9637 (18.0) | 4830 (17.7) |  |
| A-levels or equivalent | 6422 (12.0) | 3370 (12.4) |  |
| University or college | 25843 (48.2) | 13630 (50.0) |  |
| Body mass index (%) |  |  |  |
| Normal (18.5 to <25 kg/m^2^) | 20364 (38.9) | 10136 (37.1) | 0.055 |
| Underweight (<18.5 kg/m^2^) | 391 (0.7) | 122 (0.4) |  |
| Overweight or obesity (25 kg/m^2^ or higher) | 31601 (60.4) | 17026 (62.4) |  |
| Daily drinker (%) | 9146 (17.0) | 4761 (17.4) | 0.012 |
| Current smoker (%) | 1851 (3.5) | 1001 (3.7) | 0.012 |
| Menopause (%) | 26435 (49.0) | 12170 (44.6) | 0.088 |
| Hand grip strength (%) | 28.56 (10.60) | 29.77 (10.54) | 0.115 |
| Bone mineral density (g/cm^2^), Mean (SD) |  |  |  |
| Heel bone | 0.55 (0.14) | 0.56 (0.14) | 0.089 |
| Arm | 0.93 (0.15) | 0.95 (0.14) | 0.150 |
| Lumbar | 1.19 (0.20) | 1.22 (0.18) | 0.152 |
| Femur | 0.94 (0.14) | 0.96 (0.13) | 0.158 |
| Fracture history within 5 years (%) |  |  |  |
| None | 48706 (91.0) | 25110 (92.0) | 0.042 |
| Trunk | 196 (0.4) | 63 (0.2) |  |
| Other | 4642 (8.7) | 2111 (7.7) |  |
| Diabetes (%) | 3170 (5.9) | 1571 (5.8) | 0.007 |
| Insulin (%) | 427 (0.8) | 201 (0.7) | 0.007 |
| Hypertension (%) | 16959 (31.6) | 8268 (30.3) | 0.028 |
| Blood pressure medication (%) | 13355 (25.0) | 6425 (23.5) | 0.033 |
| Hypercholesterolemia | 15360 (28.6) | 7376 (27.0) | 0.035 |
| Cholesterol lowering medication (%) | 13364 (25.0) | 6369 (23.3) | 0.038 |
| Cardiovascular disease (%) | 2921 (5.4) | 1453 (5.3) | 0.003 |
| Bone disease (%) | 775 (1.4) | 302 (1.1) | 0.029 |
| Arthritis (%) | 9651 (17.8) | 4785 (17.5) | 0.007 |
| Cancer (%) | 7663 (14.2) | 3517 (12.9) | 0.040 |

SMD: standard mean difference

eTable 2. Hazard ratios of individual parameters in the risk model for osteoporosis

|  | Hazard Ratio (95% CI) | P value |
| --- | --- | --- |
| White Ethnicity | 0.58 (0.54, 0.62) | <0.001 |
| Age | 1.05 (1.05, 1.06) | <0.001 |
| Household income |  |  |
| Less than £30,999 | Reference |  |
| £31,000 to £51,999 | 1.19 (1.13, 1.25) | <0.001 |
| Greater than £52,000 | 0.9 (0.84, 0.96) | <0.001 |
| Qualification |  |  |
| Other | Reference |  |
| GCSEs or equivalent | 1.79 (1.66, 1.94) | <0.001 |
| A-levels or equivalent | 2.3 (2.13, 2.49) | <0.001 |
| University or college | 1.45 (1.35, 1.57) | 0.003 |
| Body mass index |  |  |
| Normal (18.5 to <25 kg/m^2^) | Reference |  |
| Underweight (<18.5 kg/m^2^) | 3.36 (3.14, 3.59) | <0.001 |
| Overweight or Obesity (25 kg/m^2^ or higher) | 1.39 (1.31, 1.47) | <0.001 |
| Menopause | 1.52 (1.42, 1.63) | <0.001 |
| Hand grip strength | 0.97 (0.97, 0.97) | <0.001 |
| Bone mineral density (g/cm^2^) |  |  |
| Heel bone | 0.34 (0.27, 0.43) | <0.001 |
| Arm | 0.6 (0.44, 0.81) | 0.001 |
| Lumbar | 0.11 (0.09, 0.14) | <0.001 |
| Femur | 0.07 (0.05, 0.09) | <0.001 |
| Fracture history within 5 years |  |  |
| None | Reference |  |
| Trunk (Spine & Pelvis) | 2.58 (2.41, 2.77) | <0.001 |
| Other | 1.24 (1.15, 1.33) | <0.001 |
| Diabetes | 1.51 (1.36, 1.66) | <0.001 |
| Blood pressure medication | 0.68 (0.64, 0.72) |  |
| Hypercholesterolemia | 1.15 (1.09, 1.22) | <0.001 |
| Cardiovascular disease | 1.24 (1.12, 1.36) |  |
| Bone disease | 2.15 (1.97, 2.33) | <0.001 |
| Arthritis | 1.19 (1.13, 1.25) | <0.001 |
| Cancer | 1.12 (1.06, 1.19) | <0.001 |

CI: confidence interval

eFigure 1. Illustration of dataset preparation for model development and validation


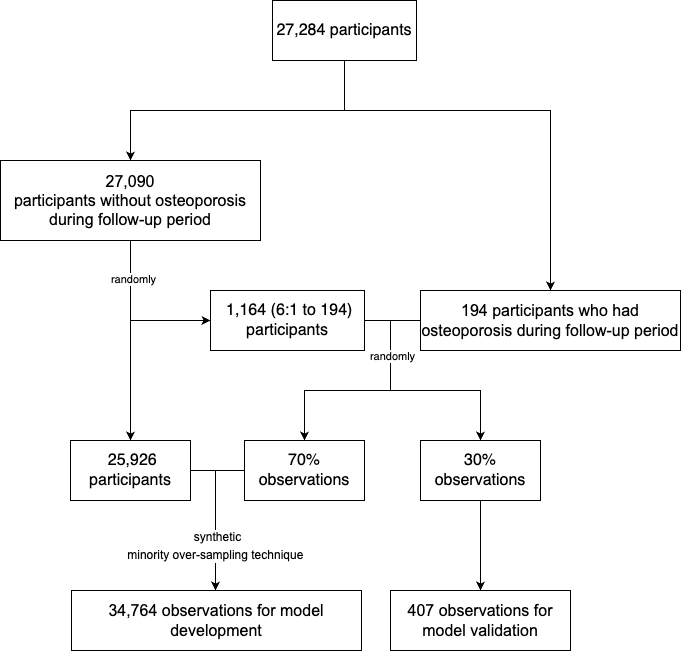


eFigure 2. Calibration of the osteoporosis self-assessment tool within the training and validation cohort


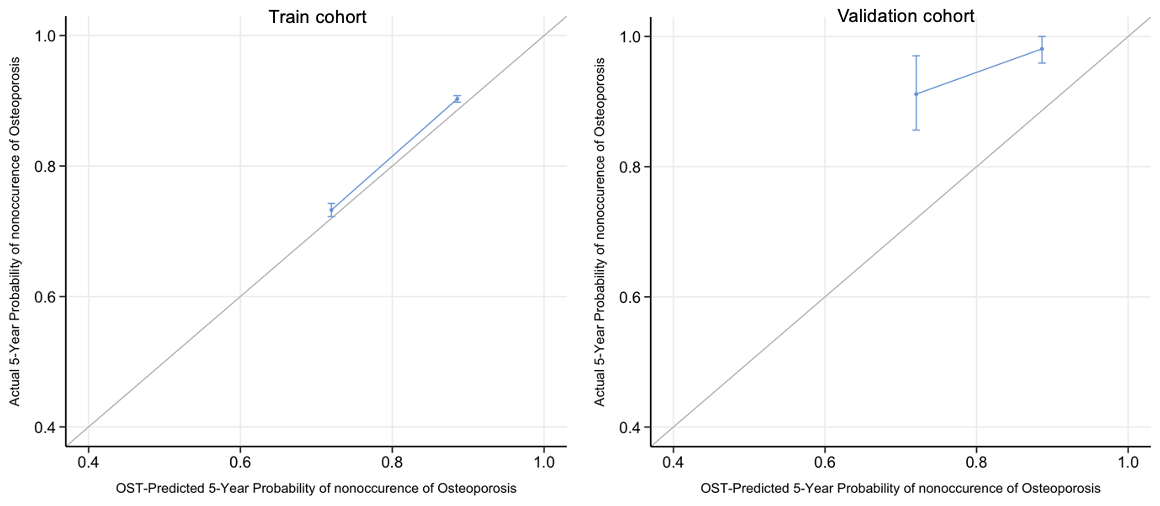


OST: osteoporosis self-assessment tool; Grey line represents perfect calibration performance.
